# Superhelical Architecture of the Myosin Filament-Linking Protein Myomesin with Unusual Elastic Properties

**DOI:** 10.1371/journal.pbio.1001261

**Published:** 2012-02-14

**Authors:** Nikos Pinotsis, Spyros D. Chatziefthimiou, Felix Berkemeier, Fabienne Beuron, Irene M. Mavridis, Petr V. Konarev, Dmitri I. Svergun, Edward Morris, Matthias Rief, Matthias Wilmanns

**Affiliations:** 1 European Molecular Biology Laboratory Hamburg, Hamburg, Germany; 2 Section of Structural Biology, The Institute of Cancer Research, Chester Beatty Laboratories, London, United Kingdom; 3 Institute of Physical Chemistry, National Centre for Scientific Research Demokritos, Athens, Greece; 4 Institute for Biophysics and Munich Center for Integrated Protein Science, Physics Department, Technical University of Munich, Garching, Germany; Brandeis University, United States of America

## Abstract

Active muscles generate substantial mechanical forces by the contraction/relaxation cycle, and, to maintain an ordered state, they require molecular structures of extraordinary stability. These forces are sensed and buffered by unusually long and elastic filament proteins with highly repetitive domain arrays. Members of the myomesin protein family function as molecular bridges that connect major filament systems in the central M-band of muscle sarcomeres, which is a central locus of passive stress sensing. To unravel the mechanism of molecular elasticity in such filament-connecting proteins, we have determined the overall architecture of the complete C-terminal immunoglobulin domain array of myomesin by X-ray crystallography, electron microscopy, solution X-ray scattering, and atomic force microscopy. Our data reveal a dimeric tail-to-tail filament structure of about 360 Å in length, which is folded into an irregular superhelical coil arrangement of almost identical α-helix/domain modules. The myomesin filament can be stretched to about 2.5-fold its original length by reversible unfolding of these linkers, a mechanism that to our knowledge has not been observed previously. Our data explain how myomesin could act as a highly elastic ribbon to maintain the overall structural organization of the sarcomeric M-band. In general terms, our data demonstrate how repetitive domain modules such as those found in myomesin could generate highly elastic protein structures in highly organized cell systems such as muscle sarcomeres.

## Abstract 2

The muscle M-band protein myomesin comprises a 36 nm long filament made of repetitive immunoglobulin–helix modules that can stretch to 2.5-fold this length, demonstrating substantial molecular elasticity.

## Author Summary

The contraction and relaxation cycles of active muscles generate substantial mechanical forces, both axially and radially, that place extraordinary stress on the molecular structures within the muscle fibers. These forces are sensed and buffered by unusually long and elastic filament proteins with highly repetitive domain structures. Myomesin is one such repetitive filament protein that is thought to form bridges between the main contractile filaments of the muscle, providing the muscle structure with resistance in the radial dimension. To investigate how the repetitive structure of myomesin contributes to muscle elasticity, we determined the overall architecture of its complete repetitive domain array using a combination of four complementary structural biology methods. Our study reveals a long, dimeric tail-to-tail filament structure folded into an irregular superhelical coil arrangement of almost identical domain modules separated by short linkers. When we applied tension to these myomesin filaments, we found they could stretch to about 2.5 times their original length by unfolding these linkers, and then return to their original state when the tension was removed. Our findings explain how myomesin might adapt its overall length in response to the changing dimensions of the contracting and relaxing muscle, so acting as a highly elastic ribbon that maintains the overall structural organization of the muscle fibers. More generally, these findings demonstrate how repetitive domain modules, such as those in myomesin, can provide elasticity to highly organized biological structures.

## Introduction

Striated myofibrils are found in skeletal and cardiac muscle cells and represent a highly organized cellular system for studying how active force can be generated while the overall structural organization of the underlying sarcomeric units is maintained. The principal protein components of myofibrils are large longitudinal filaments that include actin (thin filament), myosin (thick filament), titin, and nebulin [1]. These filaments form a well-established striated pattern of distinct zones, with the M-band at the center [2]. On activation, both substantial axial and radial forces are generated within the overall sarcomere structure [3]. To maintain a constant sarcomere volume under defined physiological conditions, these forces can lead to changes in both radial and longitudinal contour dimensions of the sarcomere. Under typical tension conditions, M-band-associated thick filaments can substantially move away from the sarcomeric center by 0.1 µm or more, which can lead to M-band-induced instability of the sarcomere [4]. Because of the presence of a stiff Z-disk architecture at the sarcomeric periphery, the amount of movement decreases with the overall sarcomere length so that the resting tension stays constant. In cardiac muscles, elastic M-band motions are thought to correlate with heart beat rate [5], rendering investigations of the underlying molecular parameters highly relevant to heart and skeletal muscle research.

To ensure the restoration of sarcomere integrity on activation, there are two principal structural compartments with elastic properties. The first section is defined by the I-band segment, which is situated between the stiff and highly interconnected Z-disk at the sarcomere periphery and the more dynamic central A-band and M-band [1],[6]–[9]. The second site for molecular elasticity is within the M-band, in which so-called M-bridges transversely connect thick filaments with each other and with titin filaments [2],. At the molecular level, M-bridges are thought to be primarily composed of myomesin (*MYOM1*), which is universally expressed, and two related isoforms, *MYOM2* and *MYOM3*, which show tissue-specific expression [12]. The three proteins share a common domain topology that is characterized by a unique N-terminal myosin-binding domain, followed by an array of fibronectin type III (Fn-III) domains and immunoglobulin-like (Ig) domains. In addition, they are capable of forming C-terminal tail-to-tail homodimers, as shown for *MYOM1* and *MYOM3* [12],[13]. Correct M-band localization of myomesin depends on the presence of the C-terminal M-band region of the titin filament [14]; myomesin, titin, and the filament protein obscurin localize to the same region [14], assigning myomesin a central role in maintaining the M-band architecture. The crucial importance of myomesin for sarcomere integrity has been shown by studies suppressing *MYOM1*, leading to disintegration of obscurin in the M-band [15]. However, in the absence of ultrastructural data at a molecular resolution, the overall organization of M-bridges, and any associated requirements for molecular elasticity, has remained largely unknown to date.

To address these questions we have made use of a previous prediction suggesting that the entire C-terminal part of the myomesin filament consists of an array of repetitive Ig domains followed by exposed α-helical linkers [16]. Here, we report the complete structure and extent of longitudinal elasticity of the entire C-terminal tail-to-tail myomesin filament My9–My10–My11–My12–(My13)_2_–My12′–My11′–My10′–My9′ (My9–My13). It folds into a superhelical architecture with almost identical Ig domain/helix modules and an estimated overall length of 360 Å in the absence of external tension. When stretched by low molecular forces <30 pN, myomesin can be extended reversibly by about 2.5-fold its original length, demonstrating that this filament can adapt its overall length to the changes in dynamic M-band dimensions that have been observed in operating myofibrils [2],[3].

## Results

### High-Resolution Structural Analysis of the C-Terminal Myomesin Array My9–My13 by X-Ray Crystallography

**Figure 1:**
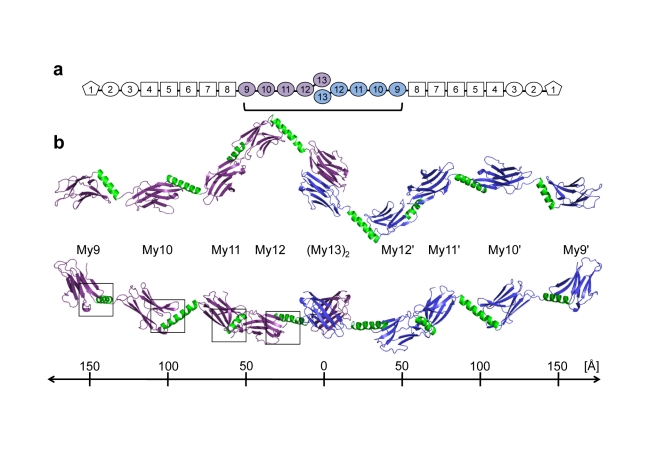
Overall filament architecture of the dimeric myomesin IgH domain array My9–My10–My11–My12–(My13)_2_–My12′–My11′–My10′–My9′. (A) Schematic representation of the complete myomesin dimer. Those My domains that have been structurally investigated are shown in violet (first molecule) and blue (second molecule). (B) Ribbon representation of the complete myomesin tail-to-tail filament structure, in two different orientations, rotated around a horizontal axis by 90°. The helical linkers are shown in green. A ruler, providing an overall length estimate of the filament, is shown below. The conserved My domain/helix interface areas, shown in Figure 2B, are boxed.

**Figure 2:**
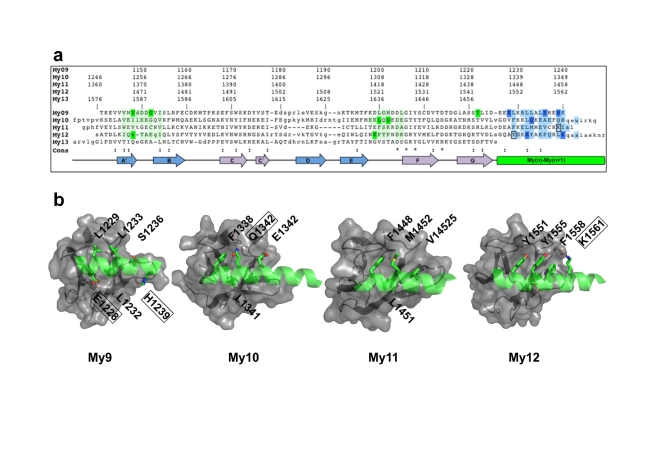
Conserved, repetitive IgH modules of My9, My10, My11, and My12. (A) Structure-based sequence alignment of My9, M10, My11, My12 IgH modules and My13. The residue numbers of each of the five sequences are indicated on top. The approximate locations of secondary structural elements are shown at the bottom (for further details see Figure S2). Highly conserved residues (:) and identical residues (*) are indicated in the consensus sequence line. Those residues that are involved in My domain/helix interfaces are highlighted in complementary colors (dark colors for specific hydrogen bonds, light colors for remaining interactions). The two residues (K1457 and Y1551) that have been mutated for SAXS studies (cf. Figure 3C) are boxed. (B) Structurally conserved My domain/helix interface areas in My9, My10, My11, and My12. Interacting helix residues are labeled; residues are boxed if involved in specific hydrogen bonds.

First, we determined the overall architecture of the complete myomesin domain array, including Ig domains My9–My10–My11–My12–My13, from crystal structures of a total of three filament fragments: the My9–My11 triplet, determined at 2.5 Å resolution; the My10–My11 doublet, at 1.9 Å resolution; and the homodimeric My11–My13 triplet at 3.5 Å resolution (Figures 1 and S1; Table 1). For further structural analysis and comparison, we also used the published crystal structure of the My12–My13 fragment [16]. Although the structure-based sequence similarity of the five Ig domains is generally below 25%, all Ig domains except My13 belong to different sub-classes of the C-set type Ig domain topology (Figures 2A and S2). A detailed comparison of the individual Ig domains My9, My10, My11, My12, and My13 is provided in Text S1.

**Figure 3:**
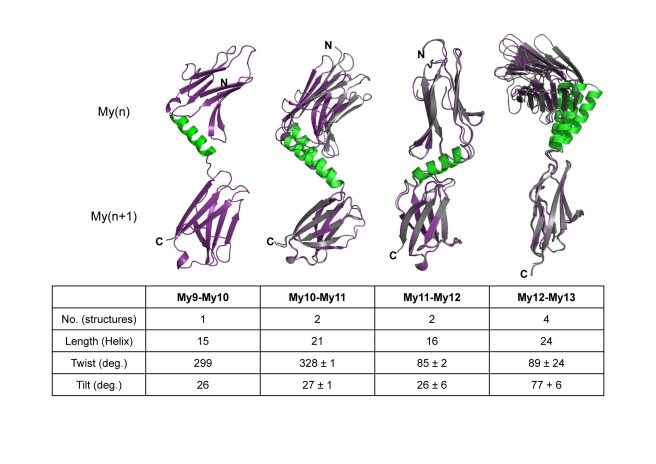
Limited flexibility of My–My domain arrangements, estimated from multiple crystal structures of identical My(*n*)–My(*n*+1) domain tandems. The number of available structures, the length of connecting helices (number of residues), and the estimated tilt and twist angles [40], defining the arrangement of adjacent My domains, are listed. The standard deviations of these angles provide an estimate of the level of My–My domain flexibility observed. Each superposition uses the C-terminal My domain as the basis for superposition. The template structure is color-coded as in Figure 1, and the remaining superimposed structures are grey. The N- and C-termini are labeled.

**Table 1:** X-ray data collection, phasing, and refinement statistics.

Myomesin Construct	My10–My11 (Native)	My10–My11 (SeMet)	My9–My11 (Native)		My11–My13 (Native)
**Data collection**					
Space group	P2_1_2_1_2_1_	P2_1_2_1_2_1_	P2_1_		P4_2_
Unit cell dimensions	a = 38.2 Å	a = 39.0 Å	a = 61.5 Å		a = 155.2 Å
	b = 74.7 Å	b = 73.8 Å	b = 41.5 Å		c = 106.3 Å
	c = 89.4 Å	c = 86.6 Å	c = 84.9 Å		
			β = 88.4^o^		
Wavelength (Å)	0.91508	0.97890	0.97699		1.0044
Resolution (Å)	23.5–1.85 (1.89–1.85)	20–2.62 (2.66–2.62)	30–2.49 (2.53–2.49)		20.0–3.50 (3.58–3.50)
*R*_merge_ (%)	4.7 (49.7)	9.9 (38.9)	11.2 (51.6)		7.6 (29.3)
I/σI	33.9 (2.7)	22.6 (5.1)	11.1 (1.8)		10.8 (2.4)
Completeness (%)	99.9 (100)	99.3 (90.2)	99.0 (89.6)		93.8 (82.0)
Redundancy	6.9 (6.6)	2.4 (1.2)	3.3 (2.9)		2.7 (2.0)
**Refinement**					
Resolution (Å)	23.5–1.85 (1.89–1.85)		25.0–2.50 (2.56–2.50)		20.0–3.50 (3.58–3.50)
Number of reflections/used for *R*_free_ calculation	22,541/716		14,571/465		29,917/1,234
*R*_work_/*R*_free_ (%)	18.9/23.9		20.9/27.1		21.7/26.6
**Number of atoms**					
Protein	1,677		2,452		9,542
Ligands	19		16		—
Solvent	164		186		—
**B-factors (Å^2^)**					
Protein	40.8		50.6		128.2
Ligands	43.2		65.1		—
Solvent	45.8		51.4		—
**Root mean square deviations**					
Bond lengths (Å)	0.006		0.016		0.014
Bond angles (°)	0.995		1.577		1.567

Values in parentheses are for the highest resolution shell.

As the available independent structures of helix-connected Ig domain doublets show only limited variation in terms of domain/domain arrangements (Figure 3), a reliable overall structural model of the complete C-terminal myomesin Ig domain array My9–My13 can be generated (Figure 1). This reveals a tail-to-tail filament structure with an overall length of 360 Å and a pronounced zigzag-type arrangement within the central, C-terminal myomesin dimerization module (My13)_2_, followed by a superhelical coil arrangement towards the two symmetrical distal ends. The right-handed superhelix is defined by almost constant twist angles of neighboring My domains of 26–27°, except the arrangement of My12–My13, which has an average twist angle of 68° and thus appears to be less regular than preceding parts My9–My10–My11–My12 of the filament (Figures 1, 3, and S3C).

Remarkably, all five Ig domains of the myomesin filament are connected by α-helices that are identical in terms of orientation with respect to the preceding Ig domain, and they form a substantial, structurally highly conserved Ig domain/helix interface with an area of 350–600 Å^2^ (Figure 2B). These interfaces involve loops that form the C-terminal tip region in each Ig domain, and residues from the first three helical turns form various specific interactions that shield the N-terminal part of each α-helix from being completely exposed. This conserved Ig domain/helix module, found in all four myomesin My domains that are followed by α-helices (My9, My10, My11, and My12), defines a new type of Ig domain topology (Figures 2B and S3). We refer hereafter to this as the “IgH” segment, which to our knowledge has not been found in any other muscle filament protein with repetitive Ig or Fn-III domains.

By contrast, we have observed neither a common interface nor a similar orientation for any of the My–My domain-connecting helices and subsequent Ig domains My10, My11, My12, and My13 (Figures 1 and S3B). As the overall geometry of the IgH segments is rigid, the limited variability in terms of arrangements of neighboring My domains originates from the diverse helix/My domain connections. Whereas long six-turn helices are found in the My10–My11 and My12–My13 connecting segments, the corresponding helices in the other two connecting segments, My9–My10 and My11–My12, are slightly shorter, with about four turns each (Figures 1 and 3). Because of the smaller length of their linkers, My9–My10 and My11–My12 show limited direct interactions in their respective Ig doublets, whereas in the other two doublets, My10–My11 and My12–My13, the neighboring My domains are too far away from each other to form an interface.

**Figure 4:**
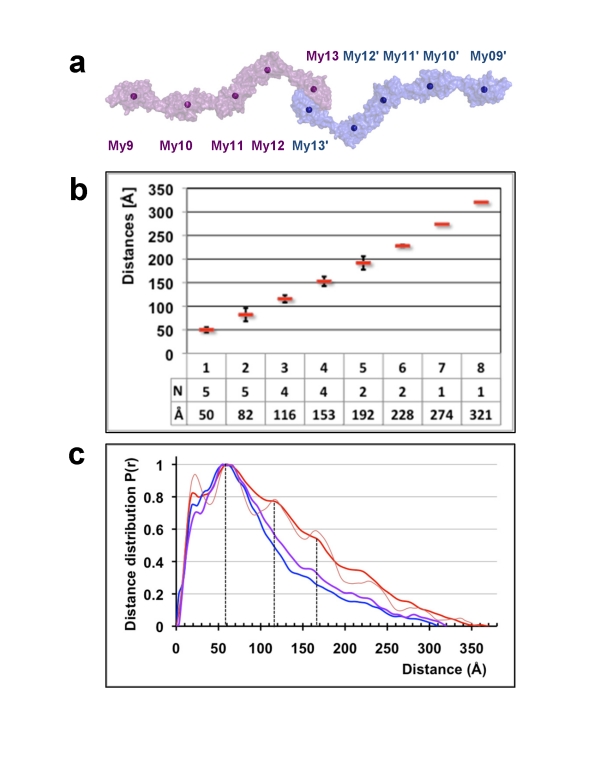
Analysis of the repetitive structural features of the My9–My13 tail-to-tail filament. (A) Surface presentation of the complete My9–My13 filament, with the centers of gravity indicated by spheres for each My domain. (B) Center of gravity distances, calculated for all My domain neighbor categories, from first (*n*, *n*+1) to eighth (*n*, *n*+8). (C) SAXS distance distribution plot of the wild-type My9–My13 filament (red) and two mutants K1457P (violet) and Y1551P (blue). The SAXS distance distribution plot calculated for the composite My9–My13 X-ray model is shown for comparison (thin red line). Matching additional maxima at about 60 Å, 115 Å, and 165 Å distances are indicated by dashed vertical lines.

The complete filament structure of the My9–My13 dimer therefore is defined by an arrangement of nine rigid bodies, comprising eight Ig domains and the central My13 dimer. A systematic distance analysis of the respective centers of gravity reveals rather narrow average distance windows, specifically for next neighbors (*n*, *n*+1, 50 Å) as well as for third and fourth next neighbors (*n*, *n*+3, 116 Å; *n*, *n*+4, 153 Å), regardless of whether they are intramolecular (within one My9–My13 monomer) or intermolecular (generated from Ig domains across the My9–My13 dimer) (Figure 4A and 4B). This distinct distribution demonstrates the highly regular arrangement of domains within the complete My9–My13 filament dimer.

### C-terminal Myomesin Filament Ultrastructure by Electron Microscopy

**Figure 5:**
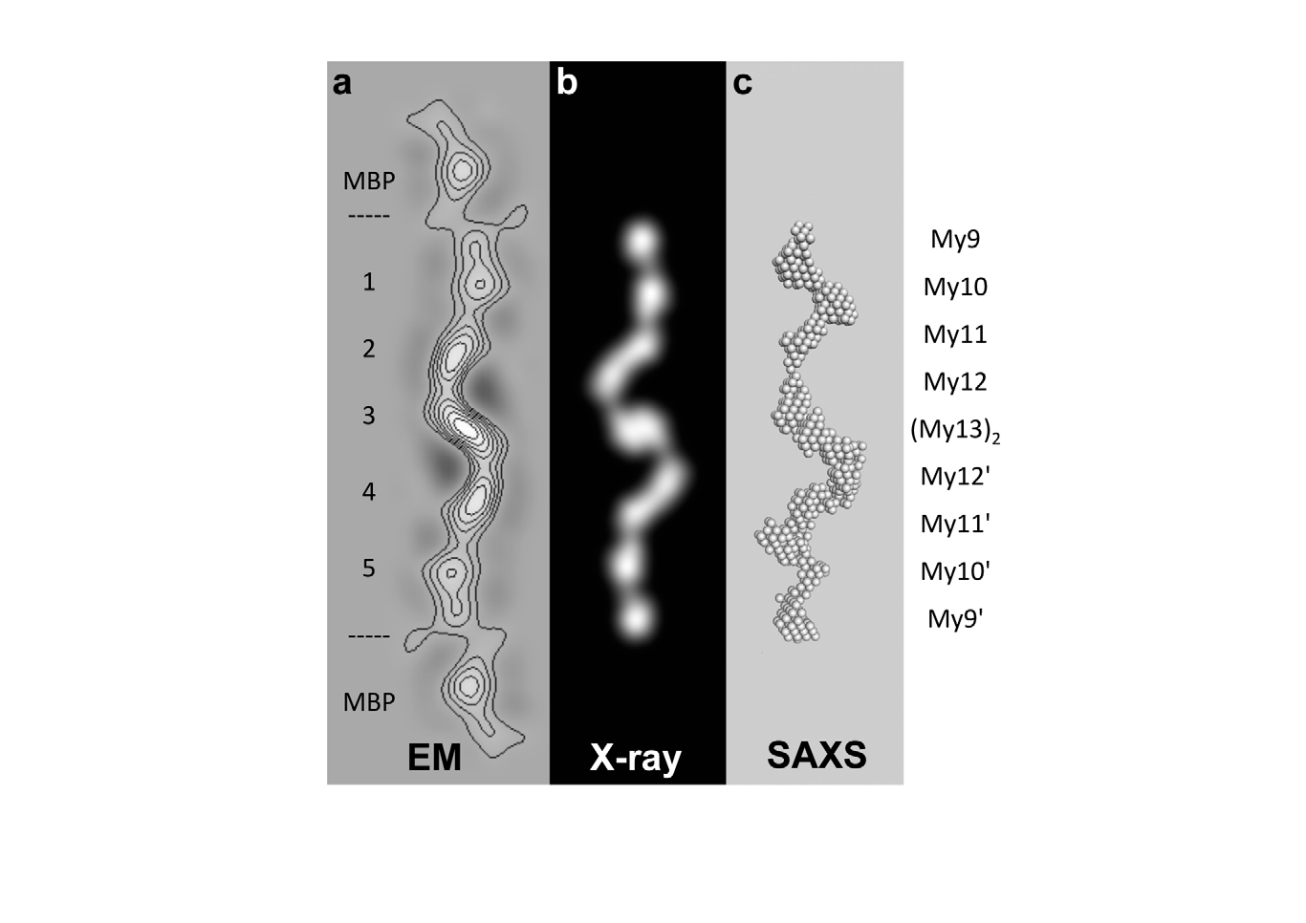
Cross-validation of the overall structure of the dimeric tail-to-tail My9–My13 filament. (A) Two-fold symmetry class average with superimposed iso-density contours. Seven stained domains can be recognized, with the two distal ones corresponding to the two terminal tagged MBP domains. The five central domains have been interpreted to be associated with the My9–My10 tandems (peaks 1 and 5), the two My11–My12 tandems (peaks 2 and 4), and the central My13 dimerization modules (peak 3). My9–My10 and My11–My12 are connected by shorter helices and therefore are less resolved as separate entities than My10–My11 and My12–My13, which are connected by longer helices (cf. Figure 1). (B) 2-D forward projection of the X-ray composite model of the dimeric My9–My13 filament (cf. Figure 1) low pass filtered to 30 Å in an orientation matching that of the EM class average. (C) Surface representation of an ab initio SAXS model of the My9–My13 filament. The My9–My13 dimer, as indicated on the right, exhibits a consistent arrangement in all data derived from EM, X-ray, and SAXS.

To independently validate the crystallographic model of the dimeric C-terminal myomesin filament, we used negative stain transmission electron microscopy (EM) (Figures 5A and S4). As it was difficult to define the precise filament ends when using a native My9–My13 myomesin fragment—probably because of limited distal flexibility coupled with the small size of each Ig domain—we fused the N-termini of each of the two myomesin My9–My13 filament protomers with maltose binding protein (MBP), which has about four times the molecular mass of a single Ig domain. This engineered version of the C-terminal myomesin filament revealed elongated molecular images about 500 Å long and 50 Å wide (Figure S4). The ends of the molecular images feature large globular densities about 50 Å in diameter, consistent with the molecular structure of MBP. Individual molecular images were aligned, classified, and averaged. A number of the resulting class averages showed 2-fold rotational symmetry, allowing the generation of averaged images in which the terminal MBP fusion and the myomesin My9–My13 filament could be unambiguously identified (Figure 5A). In these averages, the dimeric myomesin My9–My13 filament has a length that is similar to that derived from the X-ray composite model. Moreover, we could not detect any side-to-side oligomerization. This finding is further supported by complementary biophysical data for My9–My13, which do not indicate any further oligomerization (Figure S5).

The central region of the averaged EM image (Figure 5A) associated with the myomesin filament is in excellent agreement with the crystallographic My9–My13 model. The comparison suggests that the five areas of highest protein density are associated with the central C-terminal (My13)_2_ dimerization module and two flanking Ig doublets, My11–My12 and My9–My10, on both sides of the filament. In the crystal structures used to generate the crystallographic My9–My13 filament model, these two doublets are connected by shorter helices than the other myomesin/Ig domain doublets (Figure 1), which leads to an appearance of Ig domain tandems when the respective low-resolution-filtered projections are displayed (Figure 5B).

### C-Terminal Myomesin Architecture and Estimation of Molecular Flexibility by Solution X-Ray Scattering

To further characterize the overall structure of the complete My9–My13 myomesin filament in solution, we used small angle X-ray scattering (SAXS) (Figure S6). The overall parameters indicate that the particle is dimeric and that the overall shape of My9–My13, in terms of simple bodies, is best represented by a long cylinder 370 Å in length (Table 2). The resulting low-resolution shape of wild-type My9–My13 reconstructed ab initio reveals an extended coiled conformation, in agreement with both the EM and crystallographic models (Figure 5).

**Table 2:** Solution X-ray scattering data statistics.

Sample	Wild-Type	K1457P	Y1551P	Calculated
Molecular mass (kDa)^a^	130±15	115±15	110±15	129
Maximum size, *D*_max_ (Å)^b^	370±20	320±20	310±20	361
Radius of gyration *R*_g_ (Å)^b^	98±5	84±5	79±5	99
Particle excluded volume (10^3^ Å^3^)^c^	220±20	200±20	190±20	
χ, composite My9–My13 X-ray model^d^	3.0	4.0	4.2	

a Molecular mass from comparison with reference solutions of bovine serum albumin; calculated mass from My9–My13 sequence, assuming a dimer.

b Values have been obtained by indirect transformation of the scattering data using GNOM; the calculated values are from the composite My9–My13 X-ray model.

c Averaged values of multiple ab initio DAMMIN models that provide the fit with χ = 1.1.

d Discrepancy values (χ) have been calculated by CRYSOL. The ensemble of normal-mode-analysis-based models optimized by EOM provides the fit with χ = 1.2.

The computed distance distribution *p*(*r*) displays a series of maxima at distances of 60 Å, 118 Å, and 165 Å (Figure 4C), which is characteristic of elongated particles with periodic domain arrangements [17]. A comparison with the domain/domain arrangement analysis of the composite My9–My13 structure (Figure 4B) indicates that the additional peaks in the experimental *p*(*r*) most likely arise from first, third, and fourth Ig domain neighbors. We further computed a *p*(*r*) function for this composite My9–My13 crystallographic model, and indeed all maxima positions agree well with the experimental SAXS data (Figure 4C). However, we observed reduced peak sharpness in the experimental SAXS distance distribution *p*(*r*) compared with the function computed from the crystallographic model (Figure 4C). This suggests a limited flexibility in the Ig domain/domain arrangements in the dimeric My9–My13 filament, in agreement with our comparison of those Ig domain tandems for which multiple crystal structures are available (Figure 3). To take this properly into account, we applied the Ensemble Optimization Method (EOM) [18] to generate an optimized ensemble that yielded an improved fit, and the resulting *p*(*r*) function displayed peak heights proportional to the experimental *p*(*r*) (Table 2; Figure S6B).

When using My9–My13 variants in which single proline residues were introduced in two helical linkers, My11–My12 (K1457P) and My12–My13 (Y1551P), to disrupt their helical conformation (Figure 2), most of the peaks in the *p*(*r*) function were lost (Figure 4C). This was accompanied by a substantial decrease in the maximum particle size (Table 2). The data from the myomesin mutants thus show that the structural integrity of these helical linkers is essential to establish the observed defined and rigid myomesin filament architecture.

### Quantification of Myomesin Molecular Elasticity by Atomic Force Microscopy

**Figure 6:**
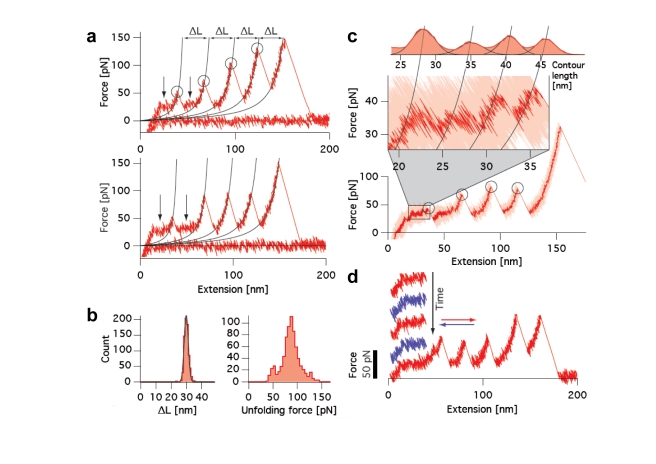
Atomic force microscopy measurements. (A) Typical force–extension traces of My9–My13 unfolding. Domain unfolding events are marked by circles. Fits to the worm-like chain model (black traces) provide contour length increases, Δ*L*, from single domains unfolding. Regions of plateau force are indicated by arrows. (B) Histograms of measured contour length increases (left, black trace is Gaussian fit) and domain unfolding forces (right). (C) Single force–extension measurement of My9–My13 (bottom) with slow pulling between 0 and 50 nm extensions. The zoom into the plateau region exhibits a substructure; black traces are worm-like chain fits. The conversion of the plateau data points to contour lengths leads to the histogram shown on top, which is fitted by Gaussians (black traces). (D) Sample traces at the plateau region when stretch (red) and relaxation (blue) cycles were introduced into the otherwise continuous stretching of My9–My13. Both stretch and relaxation cycles feature the force plateau and show no hysteresis.

The repeated structural pattern of at least four IgH modules in the C-terminal part of myomesin strongly suggests an elastic role for this segment. To test this hypothesis, we designed atomic force microscopy (AFM) experiments using an approach that was established to measure the level of molecular elasticity in the C-terminal myomesin tandem My12–My13 [19]. We created a modified version of the myomesin My9–My13 filament dimer by fusing polyhistidine tags to each of the two distal N-terminal My9 domains, and adsorbed this to a Ni-NTA-coated surface. We then probed the elasticity of the myomesin filament by adsorbing the protein chain to an AFM tip and recording the applied force versus the molecular extension. In the two sample traces shown (Figure 6A), a consistent saw-tooth pattern with a regular spacing can be observed, reflecting the sequential unfolding of individual Ig domains in the myomesin filament. The increase in contour length (Δ*L*) on unfolding can be measured with nanometer precision as 29.7 nm (Figure 6B, left) [20], which is in good agreement with the values expected for the unfolding of individual Ig domains.

At low extension and low force, however, a plateau in the force–extension curves can be observed (Figure 6A, arrows). No such plateau has been observed for Ig domain arrays in other systems investigated to date, suggesting a novel mechanism of unfolding. At high pulling velocities (typically 1 µm s^−1^), further substructures within these plateaus could not be resolved. Therefore, we performed experiments with decreased pulling velocities (10 nm s^−1^) in the critical extension range up to 50 nm, followed by an accelerated pulling velocity of 100 nm s^−1^ above an extension of 50 nm to reduce the overall experimental time. A sample trace shows that the unfolding of four Ig domains can be observed (Figure 6C, lower panel). Owing to the very low pulling velocity, the unfolding force of the first myomesin Ig domain is low and almost coincides with the plateau force. The plateau preceding this first unfolding event contains substructures, which can be seen when this region is enlarged (Figure 6C, central panel), and four distinct peaks can be resolved. A contour length histogram (Figure 6C, upper panel) reveals an average peak-to-peak distance of 6 nm. These values are in good agreement with the expected contour length increase upon unfolding of individual α-helical segments between the Ig domains. Rapid transitions can be observed between the peaks, indicating that α-helix unfolding is a rapid process close to equilibrium. This is further supported by repeated stretch (red) and relax (blue) curves within an individual plateau (Figure 6D). Both stretching and relaxing cycles exhibit the characteristic plateau. As soon as the molecule is relaxed, the helices contract back and thus act as truly elastic springs at forces around 30 pN. In summary, our AFM experiments suggest that important molecular elasticity components of myomesin originate from the helical connecting linkers between the Ig domains.

As the axial translational components for polypeptides in an α-helical conformation (1.5 Å per residue) and extended conformation (3.6 Å per residue) are known, the increase in length of a 20-residue helix on unfolding should be about 40 Å. The observed peaks at intervals of up to 60 Å when applying low pulling velocities in AFM experiments therefore match our calculations, when the straightening of substantially tilted helix orientations, as observed in the composite My9–My13 X-ray model (Figures 1 and 4), is also taken into account. Extending these calculations to the complete dimeric C-terminal myomesin filament, its estimated length with unfolded helical linkers is about 860 Å, which is almost 2.5-fold its original length, with an estimated increase in length of about 500 Å under low external forces (Movie S1).

## Discussion

The M-band is believed to be the key strain sensor in muscle sarcomeres, and important signaling events at or near the M-band may be regulated by mechanical forces originating from this region [1],[2],[21]. Members of the myomesin protein family have been identified as key bridging molecules that connect the major sarcomeric filaments in this central sarcomere segment [2]. Previous immuno-EM studies indicate that the overall orientation of myomesin has both longitudinal and transversal components, the latter being associated with the so-called M-bridges [10],[11],[22]. Although the precise orientation of different filament parts of myomesin in the sarcomere is unknown, available experimental data lead to a model in which the dimeric link via the C-terminal My13 domain in myomesin filaments is across the central M-line [13]. These data also assign myomesin an additional role in compensating the observed unbalanced filament movements with respect to the central M-line, which consequently requires a large potential of molecular elasticity.

To quantify the amount of molecular elasticity in myomesin, we applied AFM, an approach that has already been extensively used to estimate the elastic properties of the giant filament protein titin [23]. Titin filaments, however, assemble in parallel oligomeric bundles, propagating both from the Z-disk and from the M-band, and observed changes in persistence length have been estimated to be proportional to the square of the number of filaments [7],[24]. Therefore, single molecule stretching data may deviate from titin elasticity mechanisms in vivo. Accordingly, Ig domain unfolding/refolding, a process that is probably not generally reversible, has been dismissed as a mechanism for molecular elasticity under physiological conditions [23],[25],[26]. However, as there is no evidence for parallel filament bundling in myomesin (Figure S5), single molecule studies are suitable for estimating its level of molecular elasticity. In reference to the conclusions on titin, our model does not require additional unfolding of Ig domains, and, to the best of our knowledge, such reversible molecular elasticity is without precedent in any other filament system.

Our data, indicating the ability of myomesin to longitudinally extend by at about 500 Å under low external forces (Movie S1), provide a molecular model as to how myomesin could act as a highly elastic molecular strain sensor. The data presented here demonstrate that the established mechanism of molecular elasticity—reversible unfolding/refolding of highly exposed α-helical inter-domain inkers—is applicable for the complete repetitive array of IgH modules along the C-terminal part of the myomesin filament. These new findings are in agreement with previous proof-of-principle data, in which we established suitable structural biology and AFM protocols to investigate the C-terminal My12–My13 tandem [16],[19]. As the remaining domain structure of the N-terminal part of myomesin is markedly different, it is plausible that this mechanism of molecular elasticity is confined to the C-terminal My9–My13 segment of myomesin.

The length of the C-terminal myomesin filament of approximately 36 nm (360 Å) accounts for 85% of the previously estimated distance of about 44 nm between the pronounced M4 and M4′ lines in the sarcomeric M-band, which are thought to primarily consist of myomesin [2],[11],[22]. However, without more precise knowledge of the composition of these bands it remains uncertain whether bridging of the remaining distance requires some level of myomesin stretching, which would be well within our estimates of the range of myomesin extendability, or whether the part of myomesin associated with M4/M4′ is beyond the C-terminal myomesin filament My9–My13 that we have investigated. Our AFM measurements of myomesin elasticity also match the level of distance variability in the A-band observed in previous X-ray diffraction studies [3],[5].

Moreover, it will be of great interest to provide insight into how the predicted elastic properties of the C-terminal myomesin filament could functionally influence the nearby regions in myomesin that are involved in the assembly of interacting protein ligands, such as obscurin [15] and creatine kinase [27]. It remains speculative at this stage whether the coupling of mechanical forces, as they exist within the M-band, with molecular elasticity, as shown for the C-terminal part of myomesin, or with the potential regulation of kinase activity, as recently advocated for titin kinase at the M-band/A-band transition zone [21], has functional implications for interacting myomesin protein partners. Indeed, the possibility of cross-talk between mechanical stress sensing and signaling in muscle sarcomeres is an important question to be addressed. Mapping our findings on physiological stretching processes in the sarcomeric M-band is a challenging task for the future that will ultimately require phenotypic animal model studies with genetically modified versions of myomesin. Finally, it remains to be seen whether reversible filament elasticity by repetitive domain-connecting helix unfolding is unique to this M-bridge protein, or whether it also occurs in other filament systems.

## Materials and Methods

### Protein Expression and Purification

The DNA sequences (MYOM1_HUMAN) encoding myomesin domains My9–My13 (residues 1141–1666), My9–My11 (residues 1141–1447), My10–My11 (residues 1247–1447), and My11–My13 (residues 1352–1666) were amplified by PCR from existing constructs [13]. The PCR products were cloned into the pET151/D-TOPO vector (Invitrogen). The two My9–My13 single residue mutants (K1457P and Y1551P) were prepared by standard mutagenesis protocols. The MBP–My9–My13 construct was prepared by recloning the My9–My13 construct into a pETM41 vector (European Molecular Biology Laboratory). All vectors used in this study carry an N-terminal hexa-histidine tag and a tobacco etch virus cleavage site N-terminal to the myomesin encoding sequence. The constructs were expressed in *Escherichia coli* strain BL21 (DE3) CodonPlus-RP. The purification protocol included two steps: Ni-NTA affinity chromatography, followed by size exclusion chromatography (GE, Superdex 200 16/60). When required, the hexa-histidine tag was removed by 6–8 h of incubation with tobacco etch virus protease. All purified proteins were dialyzed into 25 mM Tris/HCl (pH 7.5), 150 mM NaCl, and 5 mM β-mercaptoethanol.

### X-Ray Crystallography

Crystals of myomesin protein fragments were grown by vapor diffusion, by mixing equal volumes of protein solution (8 mg ml^−1^ for My10–My11; 8 mg ml^−1^ for My9–My11; 10 mg ml^−1^ for My11–My13) and precipitant solution (0.2 M sodium nitrate, 18% [w/v] polyethylene glycol 3350, and 5% [w/w] ethylene glycol for My10–My11; 0.18 M magnesium acetate and 20% [w/v] polyethylene glycol 3350 for My9–My11; 0.1 M 2-(N-morpholino)ethanesulfonic acid [pH 6], 0.22 M lithium sulfate, and 13% [w/v] polyethylene glycol 8000 for My11–My13). X-ray data were collected on the tunable wiggler beamline BW6 (My10–My11 seleno-L-methionine [SeMet], MPG/DESY, Hamburg, Germany), on X12 (My9–My11 EMBL/DESY, Hamburg, Germany), and on BM14 (My10–My11 native, European Synchrotron Radiation Facility, Grenoble, France), and ID23-1 (My11–My13, European Synchrotron Radiation Facility). All datasets were collected at 100 K using 2-methyl-2,4-pentanediol as cryo-protectant and were integrated, scaled, and merged using the HKL suite [28]. The My10–My11 structure was determined by using phases calculated from the anomalous signal of SeMet-incorporated protein. The My9–My11 structure was solved by molecular replacement using the individual Ig domains from My10–My11 as models in MOLREP [29]. The My11–My13 structure was solved by molecular replacement using the (My13)_2_ dimer module from the My12–My13 structure (2R15) and manually fitting the remaining My11 and My12 domains. The My9–My11 structure was refined by maximum likelihood including TLS refinement, as implemented in REFMAC5 [30]. Only two residues (0.7%) are found in generously allowed regions of the Remachandran plot. The native My10–My11 and My11–My13 structures were refined using the PHENIX suite [31], implementing maximum likelihood, simulated annealing, and TLS refinement protocols and, in addition, non-crystallographic symmetry restraints for My11–My13. For the My10–My11 structure all residues are found in the most favored or additionally allowed regions of the Ramachandran plot, whereas for My11–My13, 33 residues (3.0%) are located in generously allowed regions. Details of X-ray data collection and refinement are listed in Table 1. The composite dimeric My9–My13 structure was built by superimposing the My11 domain of the My11–My13 structure (chain A) and the My11 from the My9–My11 structure. Based on the observed limited tilt/twist angle variation (Figure 3C), additional composite My9–My13 models were generated. These models led to the same conclusions when compared with the EM and SAXS data.

### Electron Microscopy

Pure MBP–My9–My13 was diluted to 10 µg ml^−1^ and applied for 1 min onto a glow-discharged carbon-coated grid and subsequently stained with 2% uranyl acetate. Micrographs were recorded using a Tecnai G2 Spirit electron microscope (FEI electron optics) at a calibrated magnification of 41,400× and an accelerating voltage of 120 kV onto SO163 Kodak films. The micrographs were digitized in a SuperCoolscan 9000 Nikon scanner at a pixel spacing of 6.35 µm, and the images were binned by a factor of three, resulting in a sampling of 4.6 Å/pixel at the specimen level. 2,075 particles were selected manually using the program BOXER in EMAN [32]. Particles were Fourier filtered using a high pass of 100 Å and a low pass of 15 Å. They were initially aligned to a reference corresponding to a streak of density with width and length similar to that of the particles. The dataset was submitted to multivariate statistical analysis using the image processing software IMAGIC [33] followed by classification and averaging. A selection of class averages acted in turn as references for alignment of the particles using the image processing software SPIDER [34]. Successive iterations of alignment, multivariate statistical analysis, classification, and averaging were carried out, and a final selection was made from a set of 100 class averages. The coordinates from the X-ray composite model were converted into density map representations using IMAGIC [33]. This map was filtered to 30 Å resolution, and a model projection image for comparison with the electron microscope data was generated by projection of the map over the full range of possible orientations.

### Small Angle X-Ray Scattering

Scattering data from purified myomesin fragments My9–My13, My9–My13(K1457P), and My9–My13(Y1551P) were measured at a concentration range of 4–15 mg ml^−1^, each with intermittent buffer solution (25 mM Tris/HCl [pH 7.5] and 150 mM NaCl), at beamline X33 (EMBL/DESY, Hamburg, Germany). The measurements were carried out at 290 K, using a sample–detector distance of 2.7 m, covering the momentum transfer range 0.10 nm^−1^<*s*<4.5 nm^−1^ (*s* = 4π sin(ϑ)/λ, where 2ϑ is the scattering angle). The data were processed using standard procedures, corrected for buffer contribution, and extrapolated to infinite dilution using the program PRIMUS [35]. The radius of gyration *R*_g_ and forward scattering *I*(0), the maximum particle dimension *D*_max_, and the distance distribution function *p*(*r*) were evaluated using the program GNOM [36]. The molecular masses of the different constructs were calculated by comparison with reference bovine serum albumin samples. The scattering patterns from the high-resolution models were calculated by the program CRYSOL [37]. Seventeen ab initio models were reconstructed from the My9–My13wild-type scattering data using the simulated annealing program DAMMIN [38]. The SAXS data statistics are summarized in Table 2. To analyze flexibility, we generated 500 models deviating from the crystal structure within root mean square deviation up to 10 Å using the low frequency normal modes [39]. EOM [18] was employed to find the mixtures of the modified structures that best fit the experimental data (Table 2), and a modified *p*(*r*) function was computed (Figure S6B).

### Atomic Force Microscopy

Single molecule force spectroscopy of My9–My13 dimers was performed on a custom-built atomic force microscope. Protein solution was adsorbed to freshly activated Ni-NTA-coated glass slides for 5–10 min and then rinsed with buffer (25 mM Tris/HCl [pH 7.5] and 150 mM NaCl) before starting the experiment. For all experiments, gold-coated cantilevers (Biolever type B, Olympus) with typical spring constants of 6 pN nm^−1^ were used. Cantilever deflection and piezo stage movement were recorded at 20 kHz. Data acquisition and analysis were performed using custom software within Igor Pro (Wavemetrics). Expected contour length increases were estimated from the difference between the contour length contribution of the end-to-end distance of the folded structure and the contour length of the corresponding sequence after unfolding. The latter is the number of amino acids multiplied by the average length contribution per amino acid, which has been determined to be 0.365±0.002 nm for our instrument using various proteins for calibration and a fixed persistence length of 0.5 nm [20].

### Accession Codes

The coordinates and experimental structure factors of the X-ray structures determined are deposited in the RCSB Protein Data Bank (http://www.rcsb.org; 2Y23, 2Y25, and 3RBS).

## Supporting Information

Figure S1 **Myomesin crystal structures used for the composite My9–My13 model.** Color codes are as in Figure 1. The resolution limit of each structure and Protein Data Bank identifier are listed. Those structures that include the C-terminal My13 domain form dimeric filament structures.
(PDF)

Figure S2 **Structural features of single Ig domains My9, My10, My11, My12, and My13.** Upper panel, topology diagrams. Secondary structural elements are labeled, and the residue numbers of their boundaries are indicated. Lower panel, ribbon diagrams of the same My domains. The color codes are defined in Figure 1. Secondary structural elements in special locations are highlighted in red. For details see text.
(PDF)

Figure S3 **Analysis of My domain arrangements.** (A) Superposition of helix/Ig domain segments, indicating variable arrangements. (B) Superposition of Ig domain/helix (IgH) segments, indicating structurally identical arrangements (Figure 2B). (C) Tilt/twist angle plot of helix/Ig domain segments from available X-ray structures (cf. Figure 3). For color definitions, see (A).
(PDF)

Figure S4 **Experimental electron microscopy data.** (A) Typical field from an electron micrograph of negatively stained MBP–My9–My13 with representative particles boxed in white. (B) Selection of aligned single particles of MBP–My9–My13. (C) Selection of four representative class averages of MBP–My9–My13. Scale bars: 20 nm.
(PDF)

Figure S5 **Biophysical characterization of the My9–My13 filament.** (A) SDS-PAGE and (B) native electrophoresis gel results. The molecular weights of some markers near the observed bands are indicated. (C) Size exclusion chromatography on an analytical Superdex 200 10/300 GL column; calibration standards are indicated. The estimate of the molecular weight by static light scattering was 143±3 kDa, associated with a polydispersity value MW/Mn = 1.002 (4%). M, markers; P, My9–My13.
(PDF)

Figure S6 **SAXS data interpretation.** (A) Experimental SAXS data: wild-type My9–My13, red; My9–My13(Y1551P), violet; My9–My13(K1457P), blue. (B) Comparison of the distance distribution functions of the wild-type My9–My13. The curves computed from the experimental SAXS data (red) and the crystallographic model (thin red line) have been taken from Figure 4C. In addition, the curve computed from the EOM-modified model is shown (thin black line).
(PDF)

Text S1 **Analysis of the myomesin Ig domain topology.**
(DOC)

Movie S1 **Molecular elasticity in the dimeric tail-to-tail myomesin My9–My13 filament.** The first part of the movie illustrates the overall architecture by zooming into and rotating the My9–My13 filament composite model and labeling the individual My domains (cf. Figure 1). The second part illustrates the collective effect that was observed in the AFM experiments (cf. Figure 6), mapped on the complete dimeric My9–My13 filament, allowing it to stretch by about 2.5 times its original length. The length estimates are indicated with a ruler. The extended model was built by straightening all Ig domains to one common orientation and by assuming unfolded helical linkers, as defined in Figures 2 and 3, with C_α_–C_α_ spacings of 3.8 Å. The estimated length for all straightened My domain modules is 290 Å, and for unfolded helical linkers 570 Å, leading to an overall length of 860 Å, which is 2.5 times the length of the X-ray-based composite My9–M13 model, in the absence of external forces (340 Å). The color codes are as in Figure 1.
(MPEG)

## Abbreviations

AFM: atomic force microscopy
EM: electron microscopy
EOM: Ensemble Optimization Method
Fn-III: fibronectin type III
Ig: immunoglobulin-like
MBP: maltose binding protein
My9–My13: My9–My10–My11–My12–(My13)_2_–My12′–My11′–My10′–My9′
SAXS: small angle X-ray scattering
SeMet: seleno-L-methionine

## References

[1] Gautel M (2011). The sarcomeric cytoskeleton: who picks up the strain?. Curr Opin Cell Biol.

[2] Agarkova I, Perriard J. C (2005). The M-band: an elastic web that crosslinks thick filaments in the center of the sarcomere.. Trends Cell Biol.

[3] Millman B. M (1998). The filament lattice of striated muscle.. Physiol Rev.

[4] Horowits R, Podolsky R. J (1987). The positional stability of thick filaments in activated skeletal muscle depends on sarcomere length: evidence for the role of titin filaments.. J Cell Biol.

[5] Pask H. T, Jones K. L, Luther P. K, Squire J. M (1994). M-band structure, M-bridge interactions and contraction speed in vertebrate cardiac muscles.. J Muscle Res Cell Motil.

[6] Kontrogianni-Konstantopoulos A, Ackermann M. A, Bowman A. L, Yap S. V, Bloch R. J (2009). Muscle giants: molecular scaffolds in sarcomerogenesis.. Physiol Rev.

[7] Tskhovrebova L, Trinick J (2010). Roles of titin in the structure and elasticity of the sarcomere.. J Biomed Biotechnol.

[8] Linke W. A (2008). Sense and stretchability: the role of titin and titin-associated proteins in myocardial stress-sensing and mechanical dysfunction.. Cardiovasc Res.

[9] Granzier H, Labeit S (2007). Structure-function relations of the giant elastic protein titin in striated and smooth muscle cells.. Muscle Nerve.

[10] Robinson T. F, Winegrad S (1979). The measurement and dynamic implications of thin filament lengths in heart muscle.. J Physiol.

[11] Al-Khayat H. A, Kensler R. W, Morris E. P, Squire J. M (2010). Three-dimensional structure of the M-region (bare zone) of vertebrate striated muscle myosin filaments by single-particle analysis.. J Mol Biol.

[12] Schoenauer R, Lange S, Hirschy A, Ehler E, Perriard J. C (2008). Myomesin 3, a novel structural component of the M-band in striated muscle.. J Mol Biol.

[13] Lange S, Himmel M, Auerbach D, Agarkova I, Hayess K (2005). Dimerisation of myomesin: implications for the structure of the sarcomeric M-band.. J Mol Biol.

[14] Musa H, Meek S, Gautel M, Peddie D, Smith A. J (2006). Targeted homozygous deletion of M-band titin in cardiomyocytes prevents sarcomere formation.. J Cell Sci.

[15] Fukuzawa A, Lange S, Holt M, Vihola A, Carmignac V (2008). Interactions with titin and myomesin target obscurin and obscurin-like 1 to the M-band—implications for hereditary myopathies.. J Cell Sci.

[16] Pinotsis N, Lange S, Perriard J. C, Svergun D. I, Wilmanns M (2008). Molecular basis of the C-terminal tail-to-tail assembly of the sarcomeric filament protein myomesin.. EMBO J.

[17] Svergun D. I, Koch M. H. J (2003). Small angle scattering studies of biological macromolecules in solution.. Rep Prog Phys.

[18] Bernado P, Mylonas E, Petoukhov M. V, Blackledge M, Svergun D. I (2007). Structural characterization of flexible proteins using small-angle X-ray scattering.. J Am Chem Soc.

[19] Berkemeier F, Bertz M, Xiao S, Pinotsis N, Wilmanns M (2011). Fast-folding alpha-helices as reversible strain absorbers in the muscle protein myomesin.. Proc Natl Acad Sci U S A.

[20] Dietz H, Rief M (2006). Protein structure by mechanical triangulation.. Proc Natl Acad Sci U S A.

[21] Puchner E. M, Alexandrovich A, Kho A. L, Hensen U, Schafer L. V (2008). Mechanoenzymatics of titin kinase.. Proc Natl Acad Sci U S A.

[22] Obermann W. M, Gautel M, Steiner F, van der Ven P. F, Weber K (1996). The structure of the sarcomeric M band: localization of defined domains of myomesin, M-protein, and the 250-kD carboxy-terminal region of titin by immunoelectron microscopy.. J Cell Biol.

[23] Linke W. A, Kruger M (2010). The giant protein titin as an integrator of myocyte signaling pathways.. Physiology (Bethesda).

[24] Houmeida A, Baron A, Keen J, Khan G. N, Knight P. J (2008). Evidence for the oligomeric state of ‘elastic’ titin in muscle sarcomeres.. J Mol Biol.

[25] Tskhovrebova L, Houmeida A, Trinick J (2005). Can the passive elasticity of muscle be explained directly from the mechanics of individual titin molecules?. J Muscle Res Cell Motil.

[26] Han J. H, Batey S, Nickson A. A, Teichmann S. A, Clarke J (2007). The folding and evolution of multidomain proteins.. Nat Rev Mol Cell Biol.

[27] Hornemann T, Kempa S, Himmel M, Hayess K, Furst D. O (2003). Muscle-type creatine kinase interacts with central domains of the M-band proteins myomesin and M-protein.. J Mol Biol.

[28] Otwinowski Z, Minor V (1997). Processing of X-ray diffraction data collected in oscillation mode.. Methods Enzymol.

[29] Vagin A, Teplyakov A (1997). MOLREP: an automated program for molecular replacement.. J Appl Crystallogr.

[30] Murshudov G. N, Vagin A. A, Dodson E. J (1997). Refinement of macromolecular structures by the maximum-likelihood method.. Acta Crystallogr D Biol Crystallogr.

[31] Adams P. D, Afonine P. V, Bunkoczi G, Chen V. B, Davis I. W (2010). PHENIX: a comprehensive Python-based system for macromolecular structure solution.. Acta Crystallogr D Biol Crystallogr.

[32] Ludtke S. J, Baldwin P. R, Chiu W (1999). EMAN: semiautomated software for high-resolution single-particle reconstructions.. J Struct Biol.

[33] van Heel M, Harauz G, Orlova E. V, Schmidt R, Schatz M (1996). A new generation of the IMAGIC image processing system.. J Struct Biol.

[34] Shaikh T. R, Gao H, Baxter W. T, Asturias F. J, Boisset N (2008). SPIDER image processing for single-particle reconstruction of biological macromolecules from electron micrographs.. Nat Protoc.

[35] Konarev P. V, Volkov V. V, Sokolova A. V, Koch M. H. J, Svergun D. I (2003). PRIMUS: a Windows PC-based system for small-angle scattering data analysis.. J Appl Cryst.

[36] Svergun D. I (1992). Determination of the regularization parameter in indirect-transform methods using perceptual criteria.. J Appl Crystallogr.

[37] Svergun D. I, Barberato C, Koch M. H. J (1995). CRYSOL—a program to evaluate X-ray solution scattering of biological macromolecules from atomic coordinates.. J Appl Crystallogr.

[38] Svergun D. I (1999). Restoring low resolution structure of biological macromolecules from solution scattering using simulated annealing.. Biophys J.

[39] Suhre K, Sanejouand Y. H (2004). ElNemo: a normal mode web server for protein movement analysis and the generation of templates for molecular replacement.. Nucleic Acids Res.

[40] Bork P, Downing A. K, Kieffer B, Campbell I. D (1996). Structure and distribution of modules in extracellular proteins.. Q Rev Biophys.

