# Supplementary material for: Superhelical Architecture of the Myosin Filament-Linking Protein Myomesin with Unusual Elastic Properties
Source: PLoS Biol. 2012 Feb 14;10(2):e1001261. doi: 10.1371/journal.pbio.1001261 (PMC3279516; doi:10.1371/journal.pbio.1001261)
Supplement: Figure S1 — Myomesin crystal structures used for the composite My9–My13 model. Color codes are as in Figure 1. The resolution limit of each structure and Protein Data Bank identifier are listed. Those structures that include the C-terminal My13 domain form dimeric filament structures. (PDF) [file pbio.1001261.s001.pdf]

Supplementary Figure 1

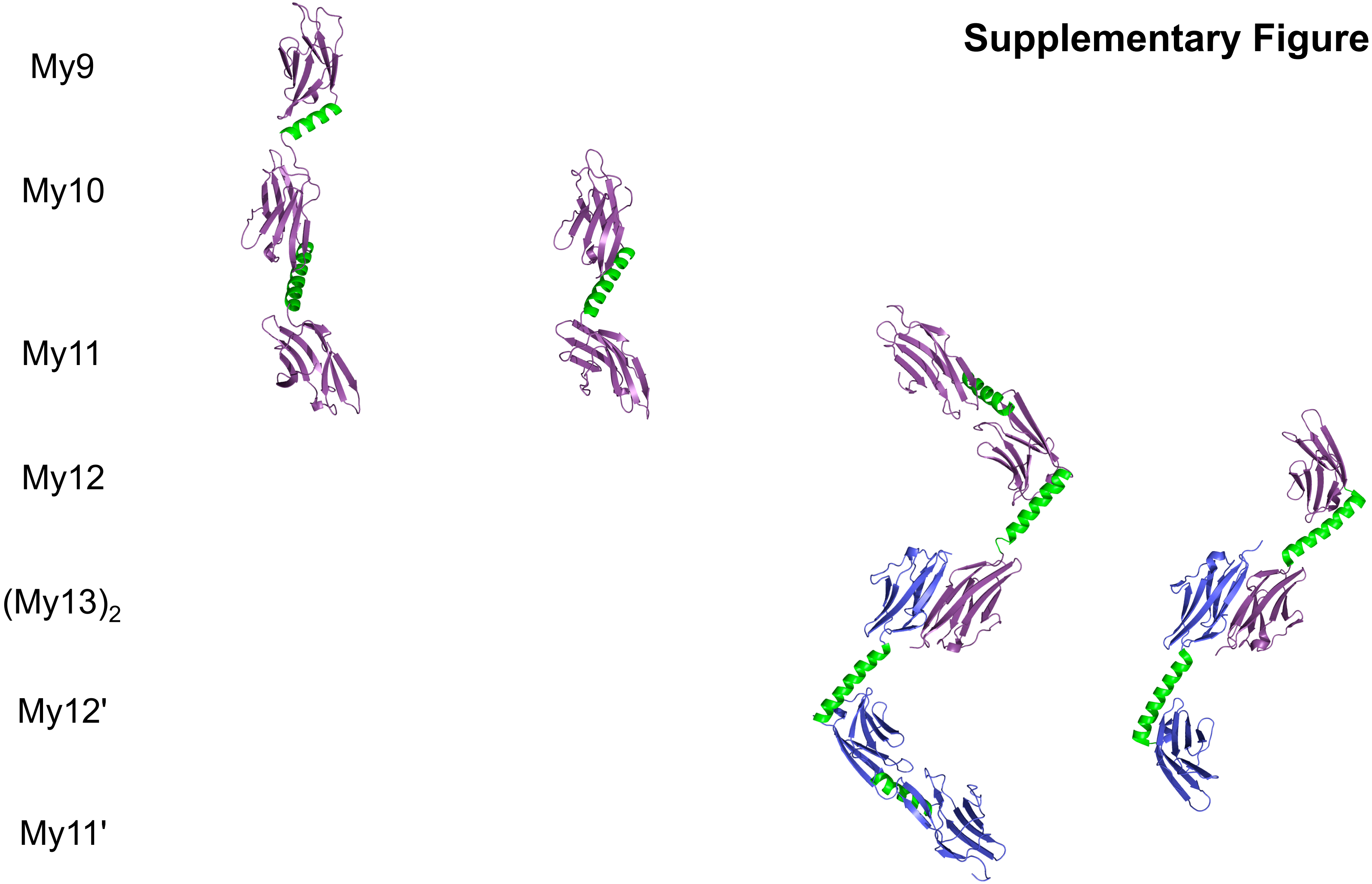

	My9-My10-My11	My10-My11	My11-My12-$(My13)_2$-My12'-My11'	My12-$(My13)_2$-My12'
Resolution	2.5 Å	1.9 Å	3.5 Å	2.2 Å
PDB	2Y23	3RBS	2Y25	2R15
