# Supplementary figures and images for: Superhelical Architecture of the Myosin Filament-Linking Protein Myomesin with Unusual Elastic Properties
Source: PLoS Biol. 2012 Feb 14;10(2):e1001261. doi: 10.1371/journal.pbio.1001261 (PMC3279516; doi:10.1371/journal.pbio.1001261)

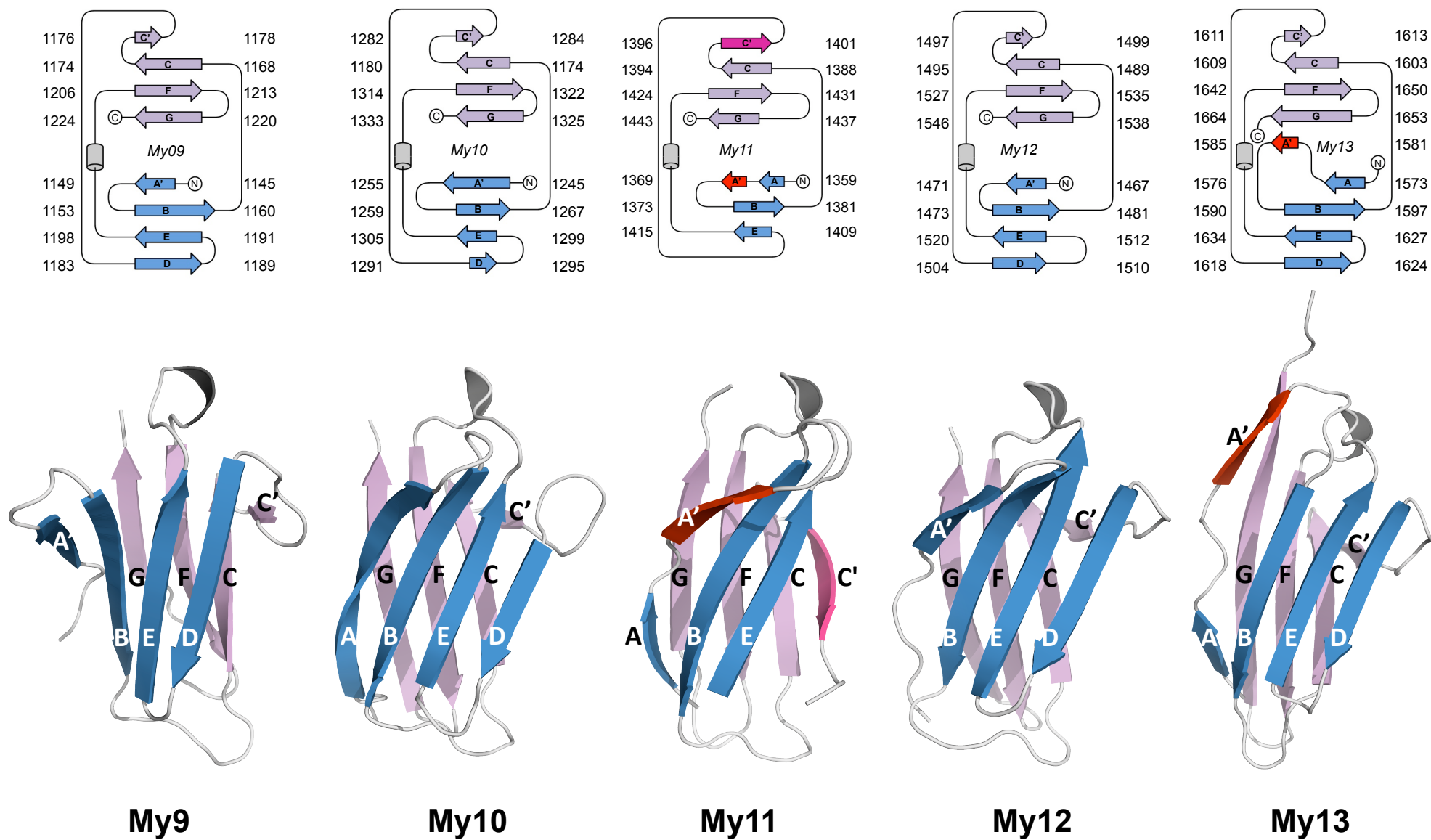

**Supplementary Figure 2**

Supplement: Figure S2 — Structural features of single Ig domains My9, My10, My11, My12, and My13. Upper panel, topology diagrams. Secondary structural elements are labeled, and the residue numbers of their boundaries are indicated. Lower panel, ribbon diagrams of the same My domains. The color codes are defined in Figure 1. Secondary structural elements in special locations are highlighted in red. For details see text. (PDF) [file pbio.1001261.s002.pdf]

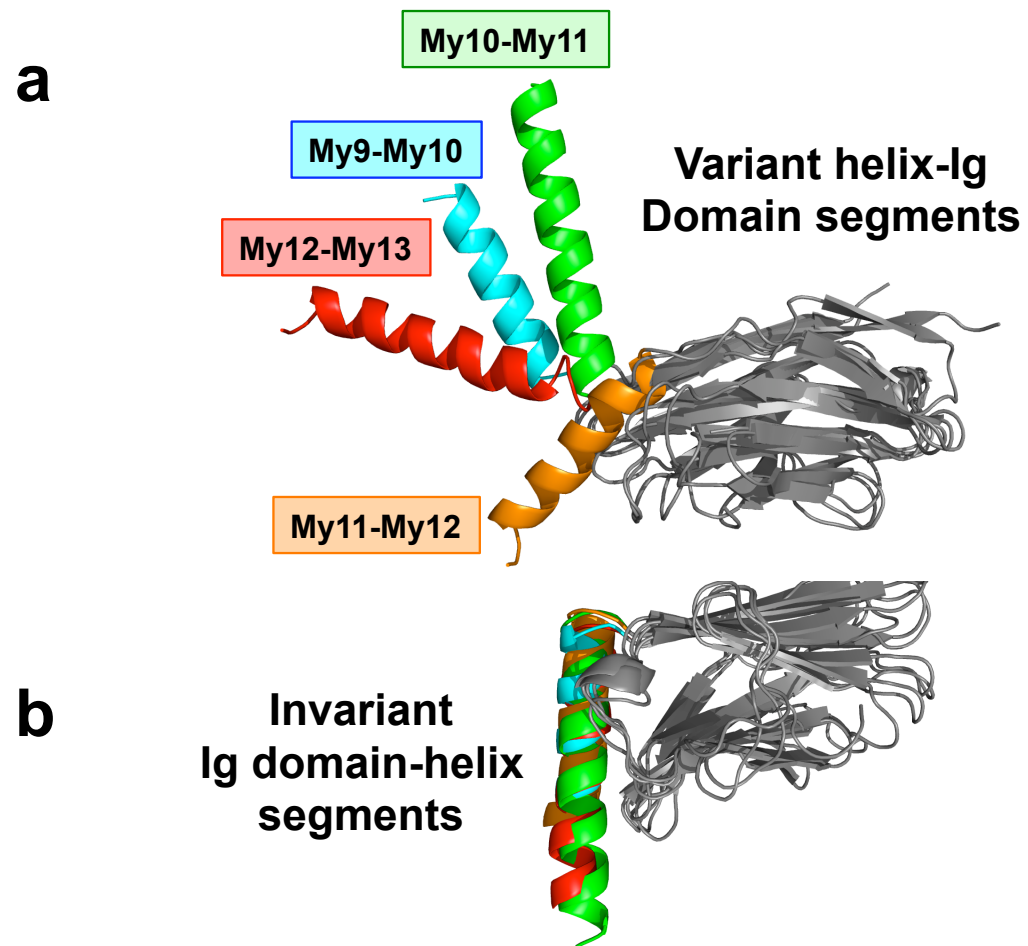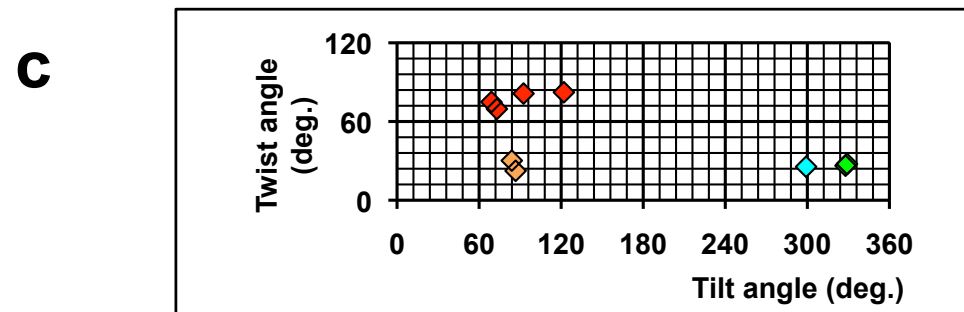

**Supplementary Figure 3**

Supplement: Figure S3 — Analysis of My domain arrangements. (A) Superposition of helix/Ig domain segments, indicating variable arrangements. (B) Superposition of Ig domain/helix (IgH) segments, indicating structurally identical arrangements (Figure 2B). (C) Tilt/twist angle plot of helix/Ig domain segments from available X-ray structures (cf. Figure 3). For color definitions, see (A). (PDF) [file pbio.1001261.s003.pdf]

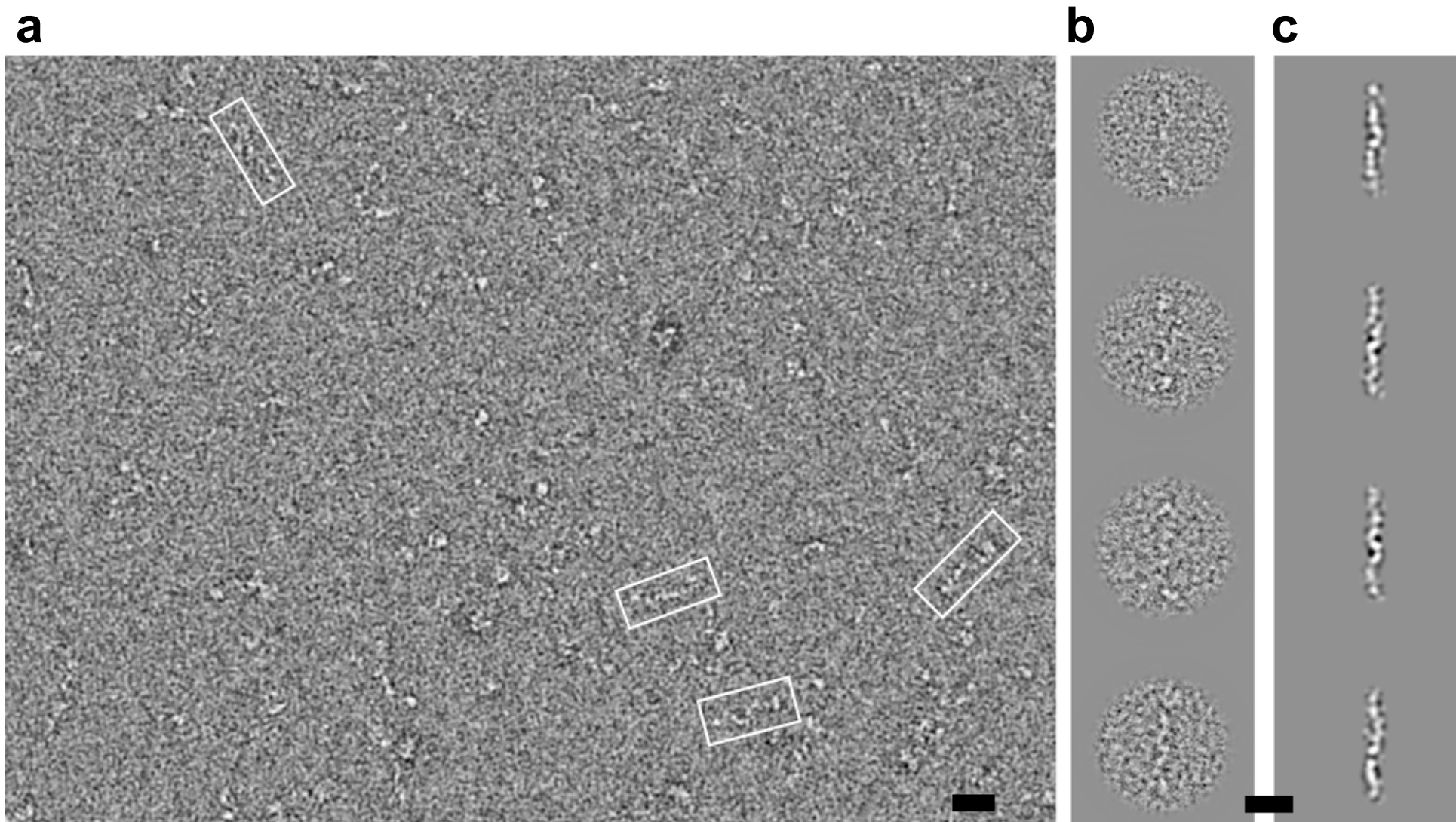

**Supplementary Figure 4**

Supplement: Figure S4 — Experimental electron microscopy data. (A) Typical field from an electron micrograph of negatively stained MBP–My9–My13 with representative particles boxed in white. (B) Selection of aligned single particles of MBP–My9–My13. (C) Selection of four representative class averages of MBP–My9–My13. Scale bars: 20 nm. (PDF) [file pbio.1001261.s004.pdf]

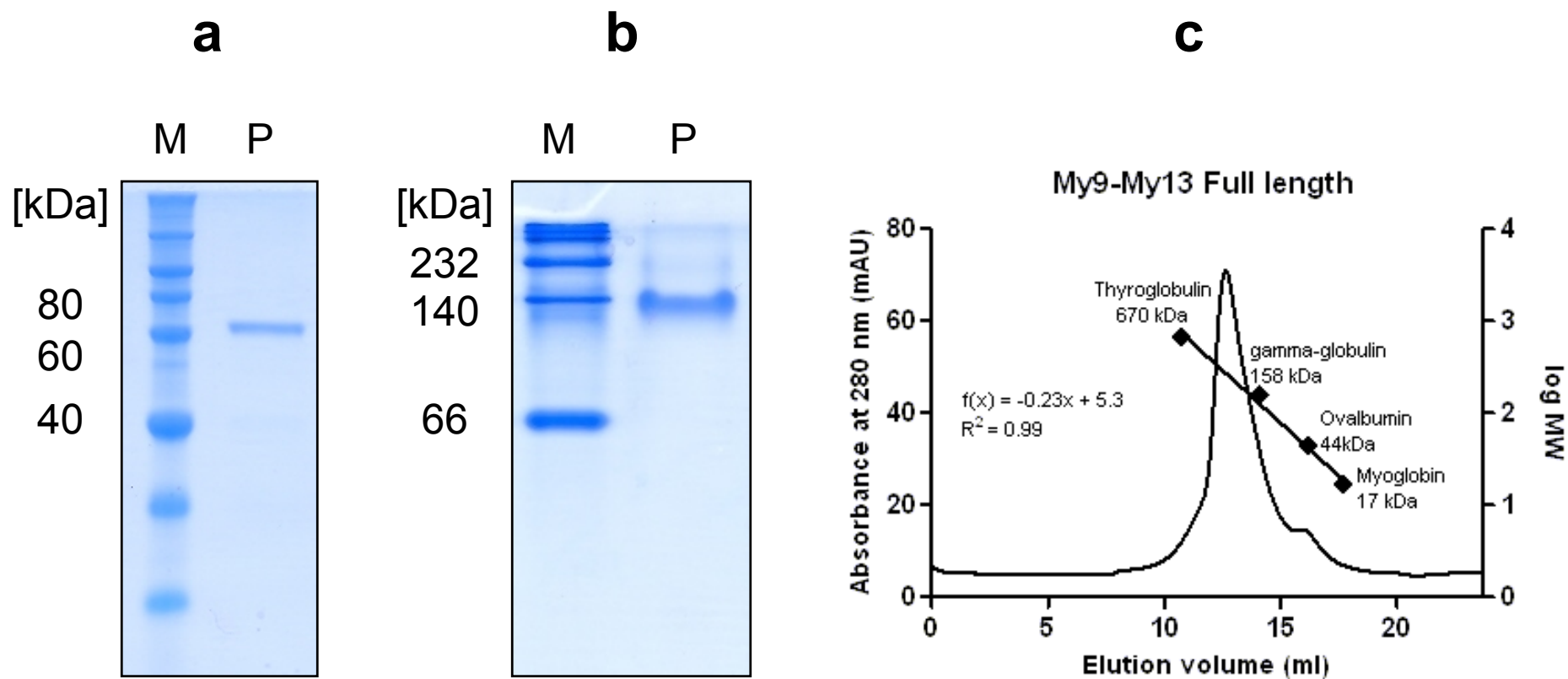

**Supplementary Figure 5**

Supplement: Figure S5 — Biophysical characterization of the My9–My13 filament. (A) SDS-PAGE and (B) native electrophoresis gel results. The molecular weights of some markers near the observed bands are indicated. (C) Size exclusion chromatography on an analytical Superdex 200 10/300 GL column; calibration standards are indicated. The estimate of the molecular weight by static light scattering was 143±3 kDa, associated with a polydispersity value MW/Mn = 1.002 (4%). M, markers; P, My9–My13. (PDF) [file pbio.1001261.s005.pdf]

**a**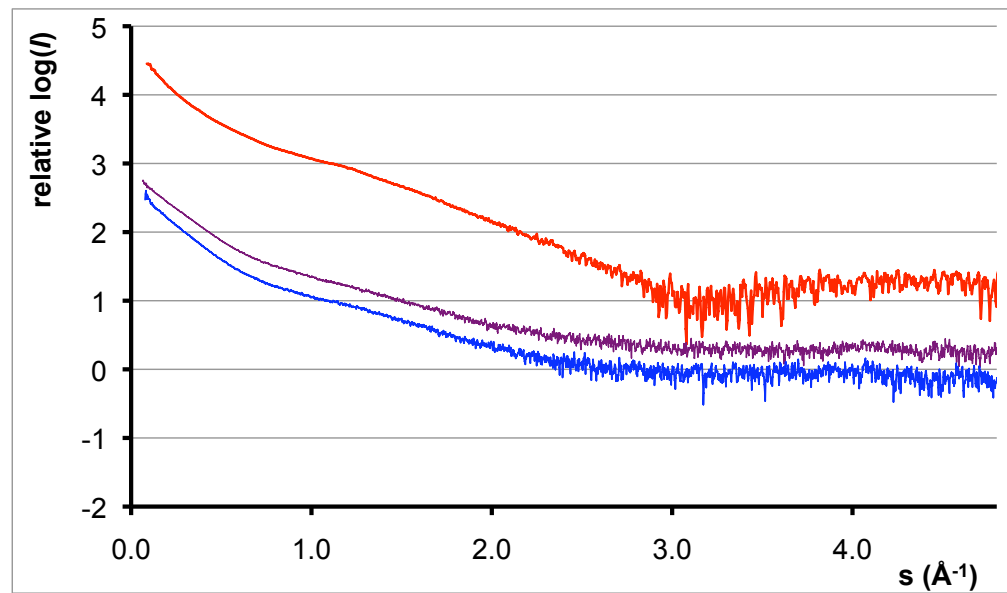**b**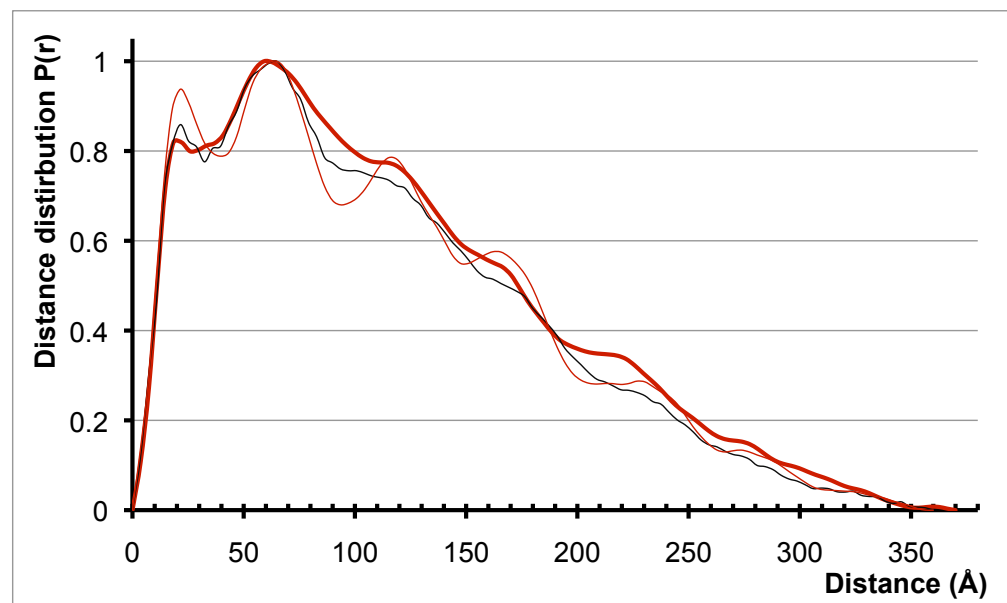

**Supplementary Figure 6**

Supplement: Figure S6 — SAXS data interpretation. (A) Experimental SAXS data: wild-type My9–My13, red; My9–My13(Y1551P), violet; My9–My13(K1457P), blue. (B) Comparison of the distance distribution functions of the wild-type My9–My13. The curves computed from the experimental SAXS data (red) and the crystallographic model (thin red line) have been taken from Figure 4C. In addition, the curve computed from the EOM-modified model is shown (thin black line). (PDF) [file pbio.1001261.s006.pdf]
