# Supplementary material for: Superhelical Architecture of the Myosin Filament-Linking Protein Myomesin with Unusual Elastic Properties
Source: PLoS Biol. 2012 Feb 14;10(2):e1001261. doi: 10.1371/journal.pbio.1001261 (PMC3279516; doi:10.1371/journal.pbio.1001261)
Supplement: Text S1 — Analysis of the myomesin Ig domain topology. (DOC) [file pbio.1001261.s007.doc]

**SUPPORTING INFORMATION**

**TEXT S1**

**Analysis of the myomesin Ig domain topology**

Based on the available crystal structures there are multiple copies for all single Ig-type myomesin domains except for My9 (only one copy), and all My domain doublets, except for My9-My10 for which only one single structure exists (**Figure S2**). Superposition of all single Ig domains My9, My10, My11, My12, My13 reveals an average rms deviation for identical Ig-type My domains of 0.68 ± 0.34 Å and a value of 1.90 ± 0.24 Å for all non-identical My domains, indicating that there are significant differences in the individual My domain structures (**Table S1).** At the level of sequence comparison, a structure-based superposition of all five My domain sequences reveals only low levels of sequence identity of < 25% for all possible pairs of myomesin My domains **(Figure 2A).** Except for My13, which has an I-set type Ig domain topology , all other Ig domains of the filament (My9, My10, My11, My12) belong to different sub-classes of the C-set type Ig domain topology**.** In two of the five Ig domains (My11, My13), the first β-strand A is split**.** Whereas in My13 the β-strand (A’) is associated with the C-terminal part of β-strand G, in My11 both β-strands A and A’ are associated with β-strand B. In the other three Ig domains (My9, My10, My12), the sequence segments that match the N-terminal part of β-strand A in My11 and My13 are too irregular in terms of overall conformation to be assigned as β-strand segments. My11 exhibits a long β-strand C’, associated with the β-strand C, whereas the subsequent β-strand D, which is observed in the other four myomesin Ig domains, is missing. In the My11-My13 triplet structure, we found the My13-mediated antiparallel C-terminal myomesin tail-to-tail filament assembly, confirming previous findings on the My13 dimerization modules .

**Table S1 Structural and sequence similarities of myomesin My domains 9-13, expressed in rms deviation and sequence similarity.**

Left column, upper row: My domain identifier, chain identifier when more than one chain is used; second left column, second upper row: PDB entry. The values of identical My domains, but from different crystal structures, are shown in bold. The structure of My11-My13 was not used in this comparison because of its limited resolution of 3.5 Å (**Table 1**).

| My ID |  | My09 | My10 | My10 | My11 | My11 | My12,A | My12,B | My13,A | My13,B |
| --- | --- | --- | --- | --- | --- | --- | --- | --- | --- | --- |
|  | PDB | 2Y23 | 2Y23 | 3RBS | 2Y23 | 3RBS | 2R15 | 2R15 | 2R15 | 2R15 |
| My09 | 2Y23 |  | 1.79 | 1.79 | 2.32 | 2.21 | 2.08 | 1.99 | 2.05 | 2.03 |
| My10 | 2Y23 | 16 |  | **0.37** | 1.86 | 1.71 | 1.48 | 1.36 | 1.84 | 1.80 |
| My10 | 3RBS | 16 | **100** |  | 1.86 | 1.67 | 1.51 | 1.32 | 1.84 | 1.80 |
| My11 | 2Y23 | 15 | 21 | 21 |  | **0.96** | 2.15 | 2.15 | 2.14 | 2.16 |
| My11 | 3RBS | 15 | 21 | 21 | **100** |  | 2.03 | 1.99 | 1.98 | 1.99 |
| My12,A | 2R15 | 13 | 24 | 24 | 21 | 21 |  | **1.06** | 2.03 | 2.03 |
| My12,B | 2R15 | 13 | 24 | 24 | 21 | 21 | **100** |  | 1.93 | 1.89 |
| My13,A | 2R15 | 18 | 15 | 15 | 24 | 24 | 19 | 19 |  | **0.68** |
| My13,B | 2R15 | 18 | 15 | 15 | 24 | 24 | 19 | 19 | **100** |  |

**Reference**

1. Pinotsis, N., Lange, S., Perriard, J.C., Svergun, D.I. & Wilmanns, M. Molecular basis of the C-terminal tail-to-tail assembly of the sarcomeric filament protein myomesin. *Embo J* **27**, 253-64 (2008).
